# The effect of intraoperative intermittent pneumatic compression on deep venous thrombosis prophylaxis in patients undergoing elective craniotomy

**DOI:** 10.3389/fneur.2024.1421977

**Published:** 2024-07-09

**Authors:** Xiang Qi, Mengrui Wang, Kang Feng, Yu Ma, Dan Zhang, Yidi Guo, Yujie Fan, Yubing Jiao, Xiaoyu Zhang, Baoguo Wang, Zhonghua Shi, Yongxing Sun

**Affiliations:** ^1^Department of Anesthesiology, Sanbo Brain Hospital, Capital Medical University, Beijing, China; ^2^Department of Anesthesiology, Peking University Third Hospital, Beijing, China; ^3^Department of Anesthesiology, Plastic Surgery Hospital, Chinese Academy of Medical Sciences, Beijing, China; ^4^Department of Ultrasound, Sanbo Brain Hospital, Capital Medical University, Beijing, China; ^5^Department of Intensive Care Medicine, Sanbo Brain Hospital, Capital Medical University, Beijing, China

**Keywords:** care, craniotomy, deep venous thrombosis, intermittent pneumatic compression, ultrasound

## Abstract

**Objective:**

Postoperative deep venous thrombosis (DVT) is commonly observed in patients undergoing craniotomy and is associated with a high incidence of pulmonary embolism and poor clinical outcomes. Herein, we investigated the prophylactic effect of DVT of intraoperative intermittent pneumatic compression (IPC) in patients undergoing craniotomy.

**Methods:**

A total of 516 patients who underwent elective craniotomy between December 2021 and December 2022 were enrolled in this study. Patients were randomly assigned to the intervention group (received intraoperative IPC) or control group (without IPC). Lower extremity ultrasound was performed on both legs before and after surgery (1 h, 24 h, and 7 days post-intervention). DVT was defined as the visualization of a thrombus within the vein lumen of the leg. Coagulation and platelet function were measured at the start and end of the craniotomy.

**Results:**

A total of 504 patients (251 in the intervention group and 253 in the control group) completed the study. Among these patients, 20.4% (103/504) developed postoperative DVT within the first week after surgery, with 16.7% occurring within 24 h. The incidence of postoperative DVT in the intervention group (9.6%, 24/251) was significantly lower than that in the control group (22.9%, 58/253, *p* < 0.001). Intraoperative IPC reduced the risk of DVT by 64.6% (0.354, 95% CI, 0.223–0.564, *p* < 0.001). There was no significant difference in coagulation and platelet function between the two groups (all *p* > 0.05).

**Conclusion:**

DVT may develop within 24 h after the craniotomy. Intraoperative application of IPC reduces the incidence of postoperative DVT.

## 1 Introduction

Deep venous thromboembolism (DVT) is a prevalent complication after general surgery, with the incidence varying from 15–80% in surgical and trauma patients (1). DVT has been associated with poor clinical outcomes, including increased morbidity and mortality (2, 3). A previous study have reported that pulmonary embolism occurred in 5–10% of patients with DVT, and the mortality rate is 9–50% (4, 5). Also, a recent study reported an incidence of DVT up to 15% in patients undergoing craniotomy (5) without preventive measures. Thus, there is an urgent need for DVT prophylaxis in these patients. Strategies of DVT prophylaxis have been widely discussed in neurosurgical patients; however, challenged by the balancing between the development of DVT and PE and the risk of catastrophic hemorrhages, there are still no recommended standard protocols for these types of patients (6–8).

Recent neurosurgical and non-neurosurgical guidelines recommend prophylactic use of anticoagulants (e.g., low-dose unfractionated heparin or low-molecular-weight heparin) to prevent postoperative DVT (9, 10). A previous study showed that prophylactic anticoagulant can reduce postoperative DVT by 58% in neurosurgical patients when no mechanical thromboprophylaxis is used (10). However, the postoperative intracranial hemorrhage associated with anticoagulant was 1.87 to3.16%, and with a 50% mortality in those who requires craniotomy for evacuation of the intracranial hemorrhage (11), limiting its application in these patients. Physical prophylaxis, such as compression stockings and intermittent pneumatic compression (IPC), has been recommended as an alternative approach for DVT prophylaxis in these patients with promising results (12, 13). IPC is a commonly used physical instrument for DVT prophylaxis in neurosurgical patients. Studies showed that IPC reduces the incidence of DVT by 64.4%^12^and could be safely and effectively used during operation (13). Prell et al. showed that intraoperative application of IPC could reduce the incidence of DVT from 26.4 to 7.3% (13). In a systematic review, Pranata et al. demonstrated a reduction in venous thromboembolism (VTE) incidence through IPC, with a decreased incidence of DVT compared to the control group (14). However, these studies primarily focused on Caucasians, while only one study (with a low sample size) was conducted in the Chinese population (15). Given the increasing number of patients receiving neurosurgery every year in China, the prophylaxis of DVT is important to improve the clinical outcomes in these patients.

Additionally, most studies reported the effectiveness of perioperative application of IPC on the development of DVT within 7 days after the craniotomy (12–15). However, in our clinical observation, the earliest DVT onset was within 24 h after surgery. Therefore, investigating the effect of intraoperative IPC on the prevention of early onset of DVT (i.e., within 24 h after operation) is important, as it could promote timely provision of the DVT intervention and could be more helpful for the physician-in-charge to decide between the dilemma of thrombosis development and intracranial hemorrhage.

In this study, we investigated the effect of intraoperative application of IPC on preventing early DVT in a larger group of neurosurgical patients. The study was performed in a tertiary medical center specializing in neurosurgical patients, with nearly 4,000 patients receiving neurosurgery annually. The primary endpoint was the difference in incidence of postoperative DVT within 24 h after surgery between patients receiving intraoperative IPC and patients without IPC. The secondary endpoint was the incidence of postoperative DVT within 7 days after surgery in both groups. In addition, the intraoperative coagulation factors and platelet function were also collected to test the predictive value of these parameters on the occurrence of DVT in these populations. We hypothesized that intraoperative IPC could reduce the incidence of postoperative DVT within 24 h.

## 2 Methods

### 2.1 Study design

This study was a single-center, randomized-controlled, prospective study trial. Ethical approval was obtained from the Ethics Committee of Beijing Sanbo Brain Hospital, Capital Medical University on Apr 12, 2023 (No: SBNK-YJ-2021-027-02). The trial was registered at: http://www.chictr.org.cn (No. ChiCTR2100053239). Informed consent was obtained from the trial participants or their legal representatives prior to the study procedure.

### 2.2 Participants

Patients who underwent elective craniotomy from December 1, 2021 to December 1, 2022, were screened for eligibility (none of the participants had received compression stockings or IPC before the surgery). The inclusion criteria were age over 18 years and the American Society of Anesthesiologists (ASA) physical status of I or II. Exclusion criteria were: coagulopathy as defined by activated partial thrombin time < 23.3 s/>32.5 s, plasma prothrombin time < 10s/>14 s, thrombin time < 14 s/>21 s, fibrin degradation products >5 mg/L or plasma D-dimer >0.55ug/mL or using anticoagulant drugs; with known vascular-related diseases within 1 year (e.g., arteriovenous thrombosis, post-varicose thrombosis syndrome, peripheral arterial occlusive disease); pregnant and lactating women. Patients who were participating in other clinical trials were also excluded. A lower extremity ultrasound was performed on both sides before the surgery, and patients were excluded if thrombosis was detected.

### 2.3 Randomization

Patients were allocated to the control group (singular number) and intervention group (even number) by referring to a computer-generated randomization table. In the intervention group, patients received intraoperative IPC during the operation. In the control group, patients did not receive IPC or compression stockings during the operation. All clinical treatments after the surgery were performed according to the clinical routine, and the physician in charge made the final decision. According to our standard clinical routine, both upper extremity venous access was selected during the operation. All surgical patients are transferred to the Intensive Care Unit (ICU) for postoperative recovery for 24 h; all surgical patients (both the intervention group and the control group) received IPC treatment immediately when transferred to the ICU, and DVT prophylaxis is performed using compression stockings when the patients were transferred to the ward.

### 2.4 Protocol of IPC

Standard calf-length IPC sleeves were placed around the calf of the patient’s lower legs before the anesthesia induction. The proximal chamber was inflated first during the intermittent compression, followed by the distal chamber in a wavelike compression. According to the previous research and our clinical experience (13). The maximum inflation pressure was 50 mmHg at a speed of 4 times per minute. The IPC was started at the beginning of the surgery and continued until the patients were discharged from the ICU.

### 2.5 Definition of postoperative DVT

Lower extremity ultrasound was performed within 1 h, 24 h and 7 days after the surgery. Both legs were assessed by the sonographer, who was not aware of the group allocation. In addition, our ultrasound results were reviewed and published individually by the same senior ultrasound doctor. DVT was defined as thrombosis detected by ultrasound in one or more veins of the legs. DVT occurred within 24 h after the surgery was used for the primary analysis.

Ultrasonography was performed with a high-resolution 5 or 7.5 MHz linear-array transducer. The deep veins were evaluated for compressibility at 1-cm intervals from the common femoral vein to the popliteal vein to the calf vein. Deep-vein thrombosis was diagnosed if the vein was noncompressible.

### 2.6 Data collection

The location and number of thrombosis detected by the ultrasound were recorded for the final analysis. Activated coagulation time (ACT), coagulation rate (CR), and platelet function (PF) were measured by Centuryclot (YKCA-2, Century Yikang Medical Technology Development Co., Ltd.) at the start and end of surgery.

Patient demographic characteristics (including gender, age, and body mass index) and medical history (including hypertension and diabetes) were collected from the patients’ electrical medical records. Data related to surgery and anesthesia, such as duration of surgery, surgical positioning, blood infusion, and drug administration, were collected at the end of surgery. Postoperative complications (including postoperative pneumonia, postoperative cerebral hemorrhage), length of hospital stay and re-operation were collected at the time of discharge.

### 2.7 Sample size calculation

Based on previous studies (13) and our clinical observations, we calculated the sample size with the assumption that the incidence of DVT was 10 and 24% in patients with and without IPC intervention, respectively. The α was set at 0.05 and β at 0.05. Considering the 30% of dropouts, a total sample of 516 patients was required.

### 2.8 Statistical analysis

The normality of the variable’s distribution was analyzed using the Kolmogorov– Smirnov test. The data were presented as mean ± standard deviation (SD), median (interquartile range, IQR), and frequency (percentage), as appropriate. Differences between the two groups were analyzed using an appropriate independent sample t-test or Mann–Whitney U test, Chi-square test or Fisher-exact test. A *p*-value <0.05 was considered statistically significant. All statistical analyses were performed using SPSS Statistics for Windows, version 25.0 (IBM Corp, Armonk, NY, USA).

## 3 Results

**Figure 1:**
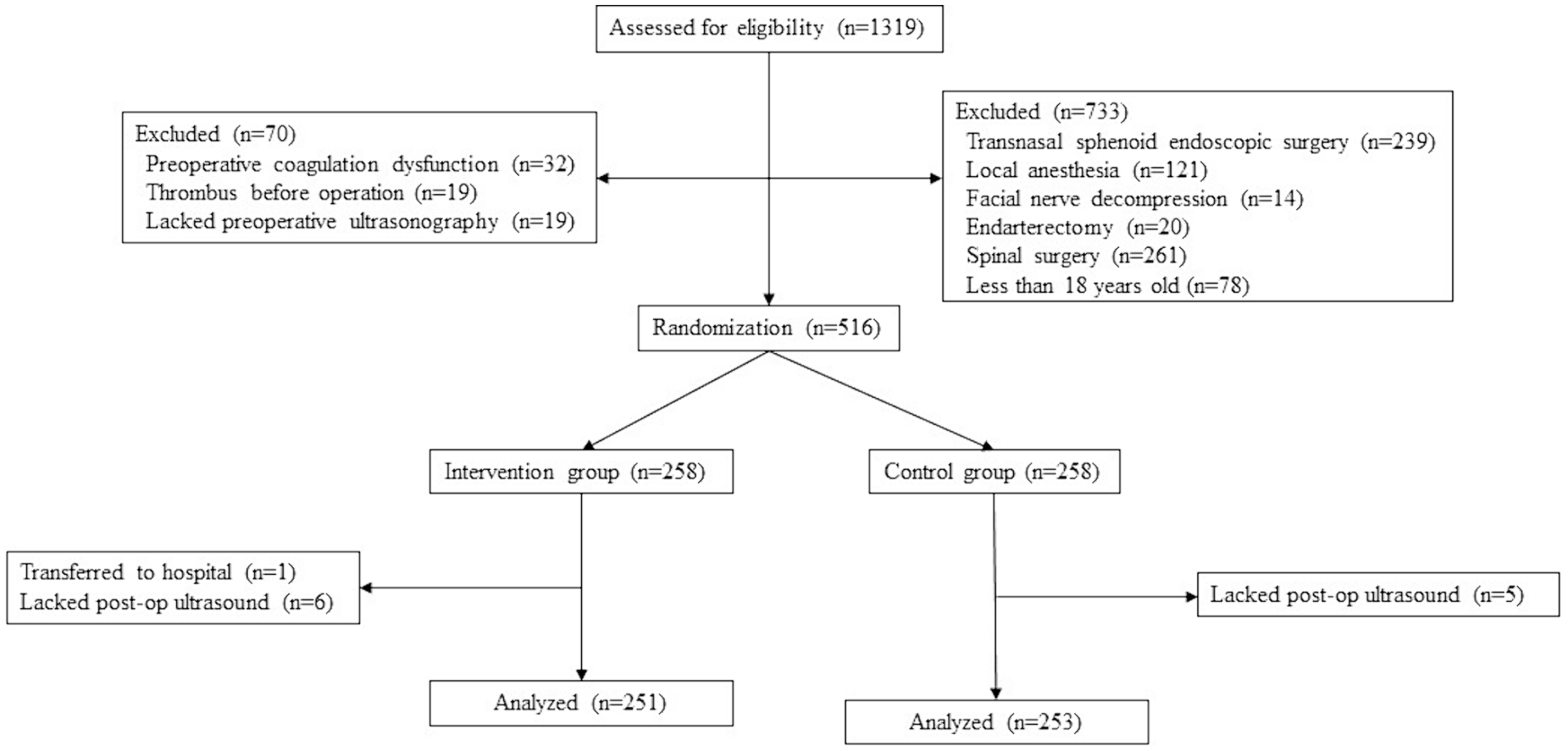
Study flow chart.

A total of 1,319 patients were screened for eligibility during the study period, and 504 patients (251 in the intervention group and 253 in the control group) were included in the primary analysis (Figure 1). One patient was removed during the study due to discharge immediately following surgery. 11 patients without lower extremity ultrasound within 24 h for practical reasons were also removed from primary analysis. Significant differences were found for sex (number of females in intervention group: 43% vs. control group: 56%, *p* = 0.002) and supine position during operation (intervention group: 55% vs. control group: 62%, *p* = 0.044) between the two groups. The histopathological classification, medical history of hypertension and diabetes, duration of operation, and fluid balance were similar between the two groups (all *p* > 0.05). More details are presented in Table 1.

**Table 1:** Demographics and baseline characteristics.

	Control group	Intervention group	*p*
**General situation**			
Age (y)*	49 (20)	48 (21)	0.130
Female sex^†^	142 (56.1)	107 (42.6)	0.002
BMI (kg/m^2^)*	24.5 (4.9)	24.8 (4.6)	0.434
**Histopathology^†^**			
Meningioma	67 (26.5)	57 (22.7)	0.339
Glioma	81 (32.0)	94 (37.5)	0.186
Schwannoma	27 (10.7)	26 (10.4)	0.672
Metastasis	12 (4.7)	12 (4.8)	0.783
Craniopharyngioma	31 (12.2)	22 (8.8)	0.134
Cavernous hemangioma	13 (5.1)	11 (4.4)	0.748
Pituitary tumor	10 (4.0)	11 (4.4)	0.799
Other	13 (5.1)	18 (7.2)	0.177
**Past medical history^†^**			
Hypertension	53 (20.9)	47 (18.7)	0.304
Diabetes	23 (9.1)	22 (8.8)	0.936
Lung cancer	7 (2.8)	6 (2.4)	1.000
Breast cancer	8 (3.2)	6 (2.4)	0.788
Use of antiplatelet drugs	14 (5.5)	13 (5.2)	1.000
Use of NSAIDs	49 (19.4)	45 (17.9)	0.732
History of surgery within three months	13 (5.1)	10 (4.0)	0.670
**Perioperative variables**			
Supine position^†^	158 (62.4)	137 (54.6)	0.044
Duration of surgery (h) *	6.0 (2.3)	6.2 (2)	0.109
Hemorrhage volume*	376 (200)	382 (250)	0.708
Fluid intake*	4,281 (1500)	4,317 (1400)	0.723

*Data are presented as median (interquartile range).^†^Data are presented as *n* (%).

### 3.1 Effect of intraoperative IPC on post-operative DVT

The overall incidence of postoperative DVT within 24 h was 16.7%, reaching 20.4% on day 7 after surgery. In addition, 81.7% of patients with postoperative DVT were detected within 24 h after the operation and 2.9% of postoperative DVT were detected in the first hour after the operation. Significantly lower rates of postoperative DVT were observed in patients receiving intraoperative IPC (9.6%) compared to patients in the control group (22.9%, *p* < 0.001). Also, this difference remained significant until day 7 after surgery (intervention group: 12.4% vs. control group: 28.5%, *p* < 0.001).

**Figure 2:**
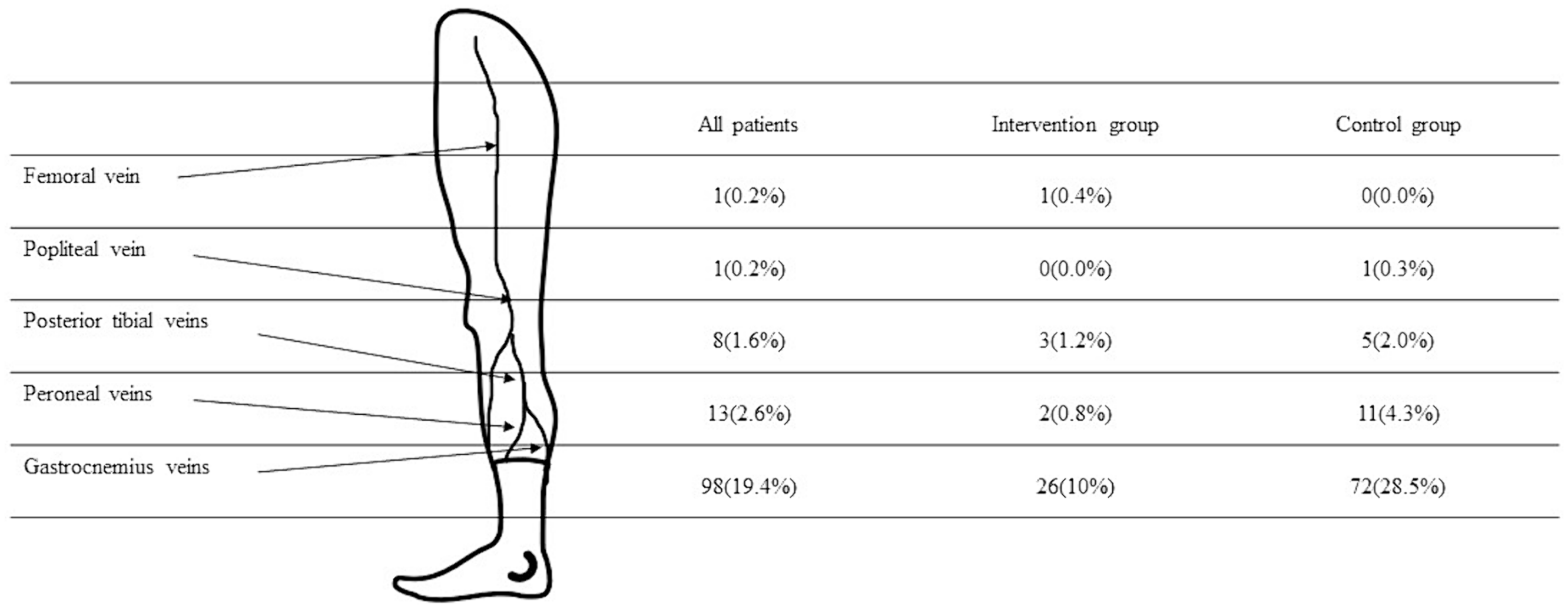
Thrombus distribution within 7 days after surgery.

Thrombosis was most frequently detected in gastrocnemius veins (94.2%), followed by peroneal veins (12.5%), posterior tibial veins (7.7%), popliteal veins (1%), and femoral vein (1%). In addition, 19 patients (18.3%) among patients with DVT had more than 2 veins involved (Figure 2). We examined each of the relevant factors and determined that intraoperative IPC, age, surgical position, and operation duration were risk factors for DVT. Logistic regression analysis of intraoperative IPC, age, position, and operation duration revealed that intraoperative IPC reduced the incidence of postoperative DVT by 63.1%(OR = 0.369, 95%CI, *p* < 0.001).

The impact of intraoperative IPC on postoperative DVT within 7 days after surgery was also analyzed, revealing a similar result (OR = 0.354, 95%CI, *p* < 0.001). In addition, different gender and positions were analyzed. Intraoperative IPC significantly reduced the incidence of postoperative DVT in different gender and supine patients (Table 2).

**Table 2:** The incidence of postoperative deep vein thrombosis stratified by different genders and positions.

	Control group	Intervention group	*P*
Female^†^	40 (28.2)	12 (11.2)	0.001
Male^†^	32 (28.8)	19 (13.2)	0.003
Supine position^†^	54 (34.2)	19 (13.9)	0.000
Lateral position^†^	18 (18.9)	12 (10.5)	0.112

^†^Data are presented as *n* (%).

### 3.2 Intraoperative coagulation parameters

**Figure 3:**
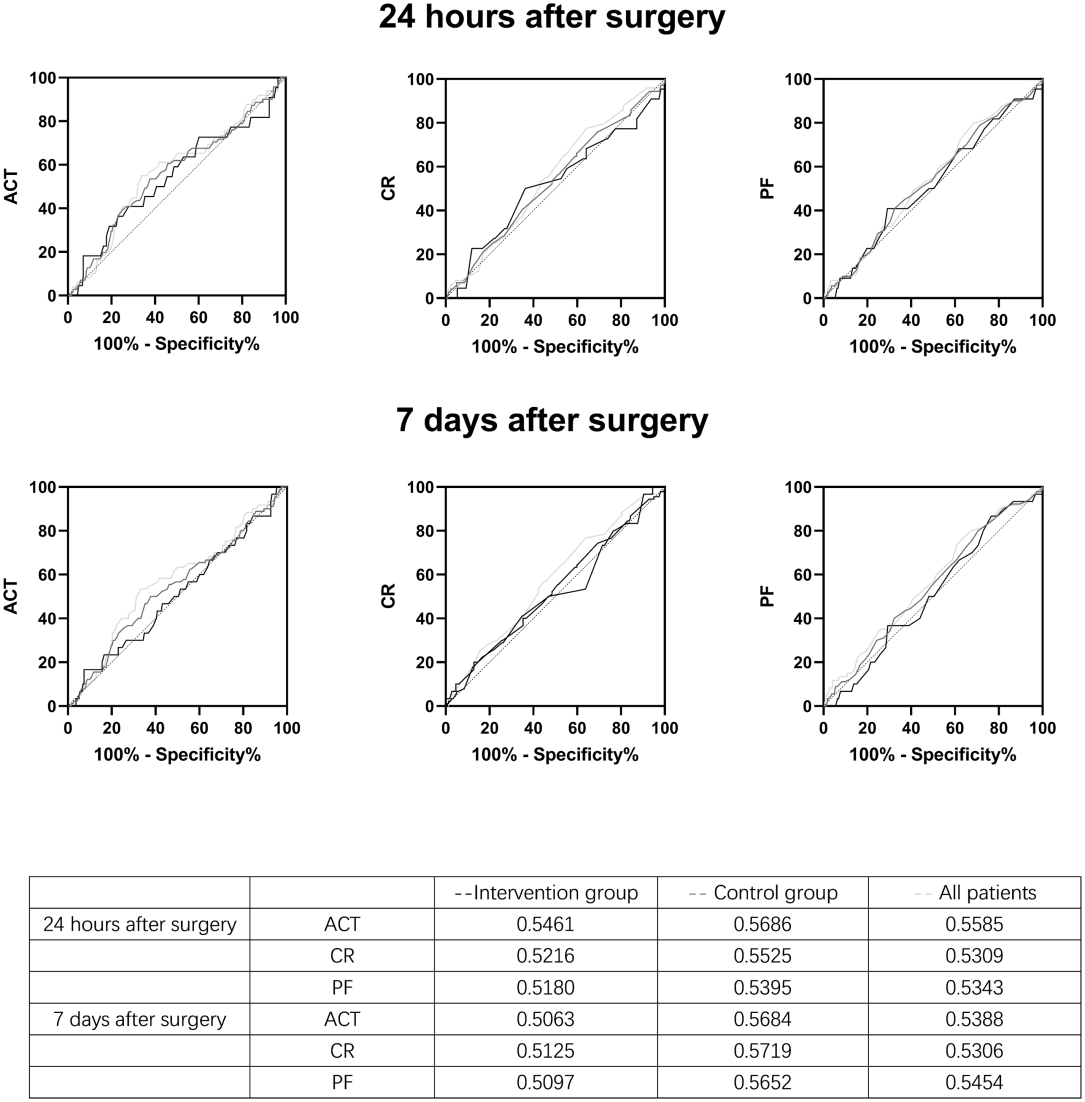
Receiver Operating characteristic (ROC) curves of the relationship between post-operative activated coagulation time, coagulation rate, and platelet function values and the incidence of thrombus 24 h and 7 days after surgery.

There was no difference in the coagulation parameters tested at the beginning and end of the operation between the two groups (Table 3). The patients were divided into a thrombus group and a non-thrombus group. Coagulation parameters measured at the end of surgery were analyzed as a predictor for the development of DVT within 24 h and 7 days after the surgery, respectively, and no significant predictive factors were found (Figure 3).

**Table 3:** The differences in activated coagulation time, coagulation rate, and platelet function between the two groups.

	Control group	Intervention group	*p*
Female^†^	40 (28.2)	12 (11.2)	0.001
Male^†^	32 (28.8)	19 (13.2)	0.003
Supine position^†^	54 (34.2)	19 (13.9)	0.000
Lateral position^†^	18 (18.9)	12 (10.5)	0.112

^†^Data are presented as *n* (%).

### 3.3 Clinical outcomes

No statistical differences in clinical outcomes were found between the intervention group and the control group (all *p* > 0.05, Table 4). There was statistical difference in clinical outcome between the thrombus group and the non-thrombus group (all *p* < 0.01, Table 5).

**Table 4:** The clinical outcomes in different groups.

	All patients	Control group	Intervention group	*p*
length of stay in hospital^*^	17 (9)	18 (7)	17 (10)	0.290
re-operation^†^	28 (5.6)	17 (6.7)	11 (4.4)	0.331
postoperative complications^†^	232 (46.0)	114 (45.1)	118 (47.0)	0.721
re-admission to the ICU^†^	34 (6.7)	22 (8.7)	12 (4.8)	0.109

^*^Data are presented as median (interquartile range).^†^Data are presented as *n* (%).

**Table 5:** The clinical outcomes in different groups.

	All patients	Thrombus group	Non-thrombus group	*p*
Re-operation^†^	28 (5.6)	14 (13.6)	14 (3.5)	0.000
Postoperative complications^†^	232 (46.0)	62 (60.2)	170 (42.4)	0.001
Re-admission to the ICU^†^	34 (6.7)	18 (17.5)	16 (4)	0.000

*Data are presented as median (interquartile range).^†^Data are presented as *n* (%).

## 4 Discussion

In the current study, the overall incidence of DVT in our cohort was 20.4% during the study period, with 16.7% of postoperative DVT occurring within 24 h after the craniotomy. Also, 3.8% of patients developed postoperative DVT >24 h to <7 days post-intervention. In addition, 81.7% of these patients with postoperative DVT development within 24 h manifested the characteristics of early onset postoperative DVT. Intraoperative IPC reduces the incidence of postoperative DVT by 64.6% (OR = 0.354, 95%CI, *p* < 0.001) within 24 h and by 63.1% (OR = 0.369, 95%CI, *p* < 0.001) within 7 days.

DVT is characterized by the formation of clots in the deep venous system. Previous studies have shown that the primarily affected veins are those in the lower leg and thighs (1). Among the three factors contributing to thrombosis, venous stasis is the most consequential; the others are vascular injury and hypercoagulability (16). Normally, the calf muscles in the legs act as pumps, and combined with the valves in the large veins, they prevent the reflux of blood and flush the blood from the lower limbs to the heart. However, for patients undergoing neurosurgery, the application of neuromuscular block drugs and prolonged immobilization during, and most of the time even after the operation, the function of calf muscles as a blood pump is obstructed. Therefore, the risk of development of DVT increases. IPC can mimic the function of the calf muscles, thus promoting the blood in the lower legs to return to the heart. Most studies reported the effectiveness of perioperative application of IPC on the development of DVT within 7 days after the craniotomy. Unlike previous studies (15), we set the primary outcome within 24 h after surgery. Our preliminary clinical observation found that many patients develop DVT within 24 h after surgery. To the best of our knowledge, no studies reported this early onset of DVT in this population. Early discovery of the DVT may help promote early intervention, which may benefit these patients. Femoral vein thrombosis was observed in one case within the intervention group. It was highly probable that the thrombus developed during the operation and entered the femoral vein under the influence of IPC. However, it was important to note that this particular case may not be fully representative, and there was insufficient evidence to definitively ascertain the cause of thrombus formation. However, this observation suggested to us that the period of surgery was likely the time when thrombosis occurred. This study also found that postoperative DVT formation was mostly concentrated within 24 h after surgery, thus suggesting that early prevention is necessary. The earliest assessment of postoperative DVT was performed in the first hour after the surgery, and there was no significant difference between the patients with and without intraoperative application of IPC. However, as the primary outcome in our study was postoperative DVT within the 24 h after the surgery, whether intraoperative IPC can prevent ultra-early postoperative DVT remains uncertain.

Most previous studies showed that the application of IPC can significantly reduce the rate of DVT both in general and neurosurgical patients (1). Prell et al. showed that IPC reduces the incidence of DVT only when each leg is considered individually (13). However, in our study, intraoperative IPC significantly reduced the rate of postoperative DVT. Our data showed that intraoperative IPC can reduce the rate of postoperative DVT by 64.6% when assessed within 24 h after surgery and by 63.1% within the 7 days post-intervention. The prevalence of postoperative DVT in neurosurgical patients ranges from 10 to 45% (1). In our cohort, the incidence of postoperative DVT was 9.6% within 24 h and 12.4% within 7 days in patients receiving intraoperative IPC, and 22.9% within 24 h and 28.5% within 7 days in patients without interventions. Our study revealed that the incidence of DVT in the Chinese population is considerable. Moreover, even with the use of physical prophylaxis, the incidence of postoperative DVT is still close to 10%. Since the vast majority of our thrombosis occurred in the intermuscular venous plexus, our hospital preferred not to use anticoagulants after measuring the postoperative intracranial hemorrhage rate and the shedding rate of the intermuscular venous thrombus. This may have been one of the reasons for the high incidence of DVT in our hospital. Unlike previous studies, our study evaluated postoperative DVT within 24 h of surgery. The use of IPC during surgery alone could prevent DVT. Also, the effect was remarkable. However, the use of IPC during surgery did not significantly prevent the occurrence of DVT 24 h later. In addition, this was somewhat different from the results of previous studies (15). Under mechanical prophylactic measures, the incidence of perioperative DVT in Chinese patients undergoing elective craniotomy for brain tumors is substantial. Without the use of IPC, the incidence of postoperative DVT at our center is in the mid-to-upper range of previous studies. We think the surgical duration of the cases we collected was relatively long. In addition, the subjects of our study were all Asian people, which may be the reason for the difference between the results of our study and previous studies. We conducted a follow-up investigation on the utilization of IPC. Both operation room nurses and surgeons reported that intraoperation IPC had no impact on the procedure of the surgery. ICU and ward nurses confirmed that intraoperation IPC did not lead to bedsores in patients, thus ensuring safety. In patients with lateral decubitus positioning, the impact of IPC was not readily apparent. This observation may be attributed to the lesser compression of calf muscles in lateral patients compared to supine patients, as well as the increased attentiveness provided by nurses to lateral patients. These factors could contribute to the outcomes observed in our study.

Hypercoagulability is one of the three major factors associated with DVT development. In patients receiving craniotomy, the surgery *per se* may generate shearing forces and cause localized ischemia, thus disrupting the integrity of the blood vessel and exposing the brain parenchyma to the plasma. Consequently, the extrinsic clotting cascade may be activated, increasing the risk of postoperative DVT (17). Therefore, intraoperative coagulation monitoring is of great significance in diagnosing the potential causes of bleeding, guiding hemostatic treatment, and predicting the risk of bleeding during long-term operation (16). In the current study, Centuryclot analysis was performed on whole blood collected from patients before and after surgical procedures (when surgical incisions were closed). Our results showed that the results of Centuryclot analysis after the surgical procedure did not effectively predict the occurrence of postoperative DVT. Moreover, we also found that intraoperative IPC did not affect the test results of intraoperative coagulation function, so the predictive value of intraoperative coagulation factors on postoperative DVT and the prevention of postoperative DVT by IPC are relatively independent.

After the patients were discharged from the hospital, we counted the duration of postoperative hospital stay, the incidence of re-operation, the incidence of a second stay in ICU, and the incidence of postoperative complications.

In the present study, intraoperative IPC did not affect the clinical prognosis of patients, which was consistent with previous studies (1). Concerns about skin injuries and reduced activity have always been the most important preventing to widespread adoption of IPC. We routinely observed the skin condition of the patients at the end of the operation and did not find any damage to the skin of the lower leg, and intraoperative IPC did not affect the early postoperative mobility of patients. This is consistent with the results of previous studies (18). Our results showed that intraoperative IPC could significantly reduce the incidence of postoperative DVT, and there was a correlation between postoperative DVT and clinical outcomes; however, our study did not directly find that intraoperative IPC can affect the clinical prognosis of patients. We consider this because our study was not based on clinical outcome as the primary indicator, so the current sample size was insufficient for comparison. In the present study, the use of IPC did not increase the incidence of secondary surgery due to cerebral hemorrhage, which makes it superior to traditional anticoagulants.

It was found that the incidence of postoperative pulmonary complications, postoperative re-operation, and postoperative re-admission to the ICU significantly correlated with postoperative lower limb venous thrombosis (*p* < 0.01). Therefore, preventing the formation of lower limb venous thrombosis can reduce the complications related to thrombosis and the incidence of postoperative pulmonary complications, postoperative re-operation, and postoperative re-admission to the ICU.

In our study, there were only 4 symptomatic cases of lower limb venous thrombosis, indicating that most thrombi were asymptomatic. This means that postoperative ultrasound is necessary, and the diagnosis should not be based only on patient’s chief complaint.

The present study has several strengths, including the systematic investigation of the impact of intraoperative IPC on postoperative DVT in a large sample of craniotomy patients. In addition, we also assessed the incidence of postoperative DVT within 1 h after craniotomy, which was reported by a few studies only. However, there are several limitations that should be pointed out. First, this was a single-center study, possibly challenging the generalizability of the data. Nevertheless, the study was performed in the largest neurological ICU in China, admitting the full spectrum of patients with brain injury. Second, the same researcher examined all the patients enrolled in our study for preoperative and postoperative ultrasound Doppler examination. Because this particular researcher could not be examined simultaneously in a short time, we excluded some patients. This situation affects the number of cases in the experimental group and thus may affect the final data analysis. Third, we only conducted lower limb ultrasound Doppler examination within 1 h, 24 h and 7 days after surgery to analyze the incidence of postoperative DVT in this time period, but did not observe the occurrence of thrombosis in more days after surgery, nor did we observe the specific time of thrombosis more intensively. Fourth, we did not conduct a comparative analysis of the benefits and drawbacks of IPC and anticoagulants; hence, we cannot furnish ample evidence to assert that IPC surpasses traditional anticoagulants. As a result, our study lacked a complete perioperative analysis of the risk of lower limb venous thrombosis. Moreover, because patients and their families often lack good compliance, the IPC was only used during surgery and postoperative anesthesia recovery. Our follow-up study will investigate whether continuous application of intermittent pneumatic compression during and after surgery long-term affects patient prognosis.

## 5 Conclusion

In patients receiving craniotomy, intraoperative application of IPC reduces the incidence of postoperative DVT by two-thirds. In addition, postoperative DVT develops as early as <24 h after the operation, highlighting the importance of timely focusing on these patients.

## Data availability statement

The raw data supporting the conclusions of this article will be made available by the authors, without undue reservation.

## Ethics statement

Written informed consent was obtained from the individual(s), and minor(s)’ legal guardian/next of kin, for the publication of any potentially identifiable images or data included in this article.

## Author contributions

XQ: Writing – review & editing, Writing – original draft. MW: Writing – review & editing. KF: Writing – review & editing. YM: Writing – review & editing. DZ: Writing – review & editing. YG: Writing – review & editing. YF: Writing – review & editing. YJ: Writing – review & editing. XZ: Writing – review & editing. BW: Writing – review & editing. ZS: Writing – review & editing. YS: Writing – review & editing, Writing – original draft.
